# Within-person fluctuations in wellbeing and task-specific work ability

**DOI:** 10.1007/s11136-017-1713-3

**Published:** 2017-10-24

**Authors:** Julitta S. Boschman, Karen Nieuwenhuijsen, Judith K. Sluiter

**Affiliations:** Coronel Institute of Occupational Health, Amsterdam Public Health Research Institute, Academic Medical Center, University of Amsterdam, PO Box 22660, 1100 DE Amsterdam, The Netherlands

**Keywords:** Multilevel Analysis, Epidemiology, Occupational health

## Abstract

**Purpose:**

To research within-person fluctuations in occupational health, work ability and wellbeing, we need new measurement strategies. We studied absolute agreement for weekly measurements of task-specific work ability and relationships between wellbeing, work demands and personal factors and task-specific work ability over time.

**Methods:**

Forty-eight Dutch academic researchers answered questions during 12 consecutive weeks. Physical and mental work demands, indicators of wellbeing and task-specific work ability in each week were measured. Intra-class correlation coefficients (ICC) for absolute agreement between task-specific work ability measures were calculated. For application in individual workers, an ICC > 0.90 was regarded as suitable. Multilevel models were used to relate both time-invariant and time-varying predictors to task-specific work ability.

**Results:**

Multiple measurements increased the reliability. Absolute agreement, however, did not reach the optimal level, except for the task ‘ability to conduct data analyses’ which had an ICC value of 0.95 (95% CI 0.91–0.98). Individuals’ leisure time physical activity (*p* = 0.03) and relational (*p* = 0.02) and social (*p* = 0.02) wellbeing were related to their average task-specific work ability. Weekly physical demands (*p* = 0.01) and personal (*p* = 0.04) and general wellbeing (*p* = 0.03) were related to weekly fluctuations in work ability.

**Conclusions:**

We demonstrated intra-individual variability in repeated assessments of task-specific work ability, pointing to the need for multiple measurements when characterizing work ability. The finding that some time-invariant and time-varying predictors can be related to the estimate of aspects of task-specific work ability and its fluctuations is helpful in understanding the dynamics of this concept.

## Introduction

A new theme in outcomes research is that we need to look at short-term, within-person fluctuations in a persons’ state [1, 2]. This approach acknowledges, for example, that individuals who are generally happy with their job may not be equally happy and engaged every day [1, 3]. The new definition of health as proposed by Huber et al. [4] fits this idea as well: the authors summarized health as a dynamic ability to adapt and self-manage in the light of social, physical and emotional challenges [5]. In other words, how a person perceives his or her health momentarily can fluctuate, depending on the challenges that he or she encounters.

When it comes to health in the context of work, work ability is a frequently used measure and is also believed to be a dynamic concept [6, 7]. Low work ability is related to sick leave [8, 9] and disability [10], and is also used to evaluate the effectiveness of interventions among the working population [11]. Tenglund [6] describes that two types of work ability can be differentiated: specific work ability in relation to a person’s normal or present job, and general work ability. The latter refers to an ability that most people have to perform some kind of work. Specific work ability is the minimal subset of manual, intellectual and social competence, together with physical, mental and social health that is required for the competence. Furthermore, for specific work ability, a person needs basic occupational virtues in order to perform the work-related tasks, given that the physical, psychosocial and organizational environment is acceptable [6]. In the present study, we focus on specific work ability.

To research outcomes of interest in the field of occupational health care, such as work demands, work ability and wellbeing [12] *dynamically*, we need new measurement strategies that capture the essence of the concept and its determinants, because in most studies these measures are determined only once or with long intervals between them. Many of the usually applied research methods remove the aspect of fluctuations in order to reduce complexity. For example, survey measures often require respondents to sum up how they felt over quite long periods of time [1]. Daily and weekly fluctuations in occupational health research are almost always ignored, and an individual approach is rarely used [13].

### The basis for studying fluctuations in work ability over time

Current measurement tools seem unable to detect individual changes and fluctuation sufficiently well. For example, the test–retest reliability (4-week interval) of the Work Ability Index has been found to be sufficient for measuring work ability at a group level [14]. However, in this study, only 25% of workers reported exactly the same score twice, whereas 44% had a lower score and 31% had a higher score, and 13% were even in a higher category (e.g. up from moderate to good work ability) and 20% were in a lower category (e.g. down from moderate to poor).

There are also examples of fluctuations in the short term from other studies on adjacent concepts that show the relevance of this approach. These include poor recovery after a busy day, hence poor sleep and increased risk of accidents the following morning [15], or the effect of more fatigue due to work on both conflicts at home and less satisfaction with work that day [16]. We consider fluctuations in responses about work ability within a worker over short periods of time as central to an understanding of the processes that underlie having poor or good work ability.

Little is known about the course of and fluctuations in work ability. Therefore, we focused in this study on addressing a possible lack of absolute agreement between measurements of task-specific work ability and formulated hypotheses that relate to a subset of processes that underlie the concept of work ability. We hypothesized that absolute agreement for two weekly measurements of task-specific work ability is low. In addition, we expected that the agreement will improve when more weekly measurements of task-specific work ability are used.

### Factors influencing task-specific work ability

In the present study, we chose to operationalize work ability in a task-specific way, in analogy with previous work [17, 18]. We assumed that task-specific work ability would be more suited for use in weekly measurements. Based on the literature [19], we chose a set of factors to study in relation to the working week. We were interested in factors that could fluctuate over a short period of time and possibly influence the perceived task-specific work ability: mental and physical load the past week and wellbeing. Basically, we tried to capture the answers to ‘How are you doing?’ and ‘What is your load at work?’ and link this to ‘How well are you able to cope with the demands at your work?’. Next to these factors, we selected factors that would fluctuate little or not at all over a short period of time but also could influence task-specific work ability: gender, age, position, number of working days per week, and the frequency and intensity of their leisure time physical activity. We hypothesized that time-invariant predictors such as gender, position, number of working days per week and leisure time physical activity are related to individuals’ usual (average) level of task-specific work ability. Furthermore, we hypothesized that time-varying predictors such as mental and physical workload and wellbeing will relate to individuals’ fluctuation in task-specific work ability.

### Research questions

We formulated the following research questions:

1. What is the absolute agreement (test–retest reliability) for two, three and twelve weekly measurements of task-specific work ability?
2. What are the relationships between time-invariant predictors (gender, position, number of working days per week and leisure time physical activity) and mean task-specific work ability?
3. What are the between-person effects and within-person effects of the time-varying predictors (weekly mental and physical workload and weekly wellbeing indicators) on fluctuation in task-specific work ability?

## Methods

### Design, study samples and procedure

We performed an observational study with a within-persons design. Academic researchers were asked to complete weekly online questionnaires, for a total of twelve consecutive weeks. We chose an interval of 1 week, as it is plausible that workers’ assessment of their work ability is most affected by the work experiences over the previous 7 days, in analogy with work functioning [20].

In October 2014, we invited the scientific personnel of five departments of an academic medical hospital in the Netherlands by email to participate in the study. All employees who spend 75% or more of their working time on research/teaching were considered ‘researchers’ and were eligible to participate. We strove to recruit 50 participants. Due to the exploratory nature of the study, we did not have the necessary information to conduct a proper sample size calculation. We based our sample size estimation on similar studies [21, 22].

As we asked human subjects questions about aspects of their work and wellbeing for several weeks, we asked the medical ethical committee for their approval of the study. They approved our study and decided that the research did not fall under the Medical Research Involving Human Subjects Act. The participants were informed in writing that completing the questionnaires was voluntary and that by completing them they gave the researchers written consent to use their information anonymously for research purposes. Informed consent was obtained from all individual participants included in the study.

At the start of the study, the participants were asked to complete a baseline questionnaire (T1). Each week after that, they were asked to complete a short follow-up questionnaire (T2–T11), which took them approximately 2–3 min. The last questionnaire (T12) took somewhat longer as more variables were included. The participants were asked to complete the questionnaire the day they received it (Thursday) or the following day. Participants who did not complete the questionnaire received a reminder on Monday and were asked to fill in the questionnaire that same day. The study was conducted from 30 October 2014 to 15 January 2015.

## Measurements

### Baseline

The baseline questionnaire (T1) contained questions on the mental and physical load of participants’ work in general (very high/high/average/low/very low). The frequency and intensity of leisure time physical activity was measured with The Dutch standard for healthy physical activity [23]. The response categories were: none or hardly/30 min at a low intensity 1–4 days a week/30 min at a low intensity at least 5 days a week/20 min at a high intensity 1–2 days a week/20 min at a high intensity at least 3 days a week. The Dutch standard is believed to be the minimum level of general physical activity to achieve health gains. The standard for healthy physical activity only discriminates leisure time physical activity from physical activity during working time. In the Netherlands, walking or cycling to and from work is considered a leisure time physical activity. The nature of the occupation of an academic researcher makes high physical activity during work unlikely.

The following demographics were asked for gender, age, position (junior researcher/PhD-student/senior researcher with a fixed contract/senior researcher with a temporary contract/principal investigator, professor/head of department/other) and number of working days per week (< 3 days/3 days/4 days/5 days).

### Weekly questions

All questionnaires asked about task-specific work ability during that week (primary outcomes) and the mental and physical load of the work and wellbeing that week (predictors). Task-specific work ability questions included the following [17]: ‘How do you rate your work ability this week with respect to the following activities: addressing meetings; writing, addressing e-mail, reading (performing general computer work); data management and analysis; performing research activities, such as lab or fieldwork; teaching (preparation and actual teaching)’. Response categories were as follows: good (8)/more than sufficient (7)/sufficient (6)/just sufficient (5)/just insufficient (4)/insufficient (3)/more than insufficient (2)/not able to perform activity (1)/did not occur this week.

The questions on mental and physical workload were as follows: ‘How do you rate the mental/physical demands of your work this week, including today?’ [very high (5)/high (4)/average (3)/low (2)/very low (1)]. The questions on mental and physical workload were general and participants were expected to average their demands over a week. We believed it was not feasible to define mental demands in the context of specific tasks, in particular as we asked for more than tasks than were eventually analysed.

Four indicators of wellbeing (personal, relational, social, general) were measured with the Outcome Rating Scale (0–100, 100 indicating best wellbeing) [24]. The indicators were clarified to the respondents as follows: personal wellbeing: individual wellbeing; relational wellbeing: wellbeing in the context of family, intimate friends; social wellbeing: wellbeing in the context of work, social contacts; general wellbeing: overall wellbeing.

### Last questionnaire

In T12, questions about the average mental and physical load of their work during the previous 3 months (very high/high/average/low/very low) were asked, whether leisure time physical activity had changed during the previous 3 months (no, not at all or hardly/yes, more physical activity/yes, less physical activity), and the number of days off and/or holidays taken during the previous 3 months (some occasional days off/a whole week off/2 weeks or longer off/none).

## Analysis

Descriptive statistics were presented as percentage, mean, standard deviation (SD), and range. Leisure time physical activity was recoded into ‘Dutch standard for healthy physical activity’ ‘not achieved’ (no or hardly any physical activity) or ‘achieved’ (one or more answers in the other answer categories). Position was recoded into ‘junior position’ (junior researcher/PhD student) and ‘senior position’ (all other categories). Number of working days per week was recoded into ‘5 working days per week’ and ‘4 working days or fewer per week’.

### Absolute agreement

To assess the absolute agreement (test–retest reliability) of task-specific work ability from one time point to another, we calculated intra-class correlation coefficients (ICC) for absolute agreement for the first two, the first three and all twelve measurements. For application in individual workers, we used the common requirement of an ICC value of 0.90 for application in individual workers [25].

### Predicting mean task-specific work ability and fluctuations

Data were eligible for modelling within-person fluctuation (i.e. within-person variation or intra-individual variability) when data from one respondent were available for at least 9 weeks, including the first and the last measurement. We did not expect a systematic within-person change in this time period as no specific intervention was offered to the workers, and we prepared the analysis accordingly. Longitudinal data allow to assess within-person associations, but also provide information about cross-sectional, between-person associations (e.g. relationships among individual differences in overall levels in addition to daily levels of mental and physical workload and wellbeing).

For outcomes in which people show within-person fluctuation over time rather than systematic change over time, random time slopes for individual differences in change will not be relevant for describing patterns of outcome variance and covariance over time and this is where alternative covariance structure (ASC) models are more useful [2]. Therefore, we chose the ACS structure for modelling fluctuation. We used the statistical approach for modelling fluctuations over time as described by Lesa Hoffman [2] and refer to her book for a more in-depth explanation of modelling within-person fluctuation.

We modelled within-person fluctuation of work ability over time by means of general linear mixed models using maximum likelihood estimation. Our first step was to find a model that best matched the outcome variance and covariance over time so that subsequent predictor effects would be tested as accurately as possible. We tried unstructured, compound symmetry and AR1 and chose compound symmetry as this had the smallest AIC (Akaike’s Information Criterion) and BIC (Schwarz’s Bayesian Criterion). To see whether there was a systematic within-person change in task-specific work ability, we checked the omnibus F-test of mean differences across weeks.

Our second step was to assess the relationships between time-invariant predictors (person-level or level 2 predictors) and mean task-specific work ability. This model for the means (fixed effects) concerns how the outcome (work ability) will vary as a function of values on the predictor variables. We included the following predictors as fixed effects: gender (coded such that male = 0), position (junior position = 0), number of working days per week (5 days per week = 0) and leisure time physical activity (insufficient physical activity = 0). These predictors were considered not likely to change over the course of the study. The variance in task-specific work ability due to person mean differences (ICC) was calculated. In addition, the proportional reduction in the random intercept variance relative to the empty means, random intercept model was calculated (pseudo R^2^). Because no effects related to time were needed in the model for the means, the only individual effect to be predicted at the within-person level is the intercept [2].

Our third step was to assess effects of time-varying predictors (longitudinal or dynamic or level 1 predictors) on fluctuation in task-specific work ability. Time-varying predictors are usually composed of two sources of variation. In our example, some people feel just “less well” than others, but it could be worse on some days than others. Weekly low wellbeing will therefore contain systematic between-person variation as well as within-person variation. These two sources of variation have differential effects on the outcome—a between-person effect and a within-person effect, respectively.

We simultaneously included mental and physical workload in a model and wellbeing indicators in another model. We used person-mean centering to centre those time-varying predictors. We were interested in the effects of mental and physical workload and variables for wellbeing in themselves [26] and therefore assumed that the within-subject effects of mental and physical workload and variables on wellbeing were fixed (i.e. that everybody gets the same effect) and that the main effects were linear and additive.

Between-person variation was shown by the variation of the person means; within-person variation is shown by the deviation of each occasion from the person mean.

IBM SPSS Statistics for Windows Version 22.0 software was used to analyse the data. Statistical significance was set at an alpha level of 0.05.

## Results

The data of all 48 academic researchers were used to answer research question 1. The number of respondents varied from week to week, with the lowest number of respondents in week 7 (n = 34). For 40 participants (32 females and 8 men, see Table 1 for their demographics), data for at least 9 weeks were available and these data were used to answer research question 2 and 3.

**Table 1:** Demographics of the university employees who participated for at least 9 weeks in the study

Variable		*n*
Gender	Female	32
	Male	8
Age (mean, range)	38 years (24–56)	
Function	PhD student	18
	Senior researcher, fixed contract	5
	Senior researcher, temporary contract	6
	Principal investigator, professor	5
	Other	6
Working days per week	5 days per week	22
	4 days per week	16
	3 days per week or less	2

At baseline, almost three-quarters indicated that they perceived the average mental load of their work in general as high of very high. As expected, more than 80% of the researchers reported that they perceived the physical load of the work as low or very low. At the end of the study, the majority (58%) of respondents reported that they perceived the mental load of their work during the previous 3 months as very high or high. Physical load during the previous 3 months was perceived as average, low or very low by almost all respondents (93%).

At the end of the study, three-quarters of the respondents reported that their leisure time physical activity had not changed over the previous 3 months. Five respondents reported more and four reported less physical activity. During the 3 months, 30% took some occasional days off and 73% took 1 or 2 weeks off.

With respect to the tasks ‘performing research activities, such as lab or fieldwork’ and ‘teaching’, more than two-thirds of the participants reported that they had not performed those tasks. As analyses were not possible due to a lack of data for these outcomes, we analysed only the outcomes ‘the ability to address meetings’, ‘the ability to perform general computer work’ and ‘the ability to conduct data analyses’. As expected, the omnibus F-test showed no significant changes over time.

### Absolute agreement for two, three and twelve weekly measurements of task-specific work ability

The absolute agreement parameters (ICCs) of three questions on task-specific work ability were 0.34 (95% CI 0.23–0.48) (‘addressing meetings’), 0.41 (95% CI 0.30–0.56) (‘addressing email’) and 0.62 (95% CI 0.46–0.78) (‘conducting data analyses’), indicating substantial variation over weeks.

For the question regarding task-specific work ability ‘to address meetings’, the test–retest reliability of the average of the first two measurements was found to be 0.65 (95% CI 0.36–0.81). When the consecutive third measurement was added, the reliability increased to 0.71 (95% CI 0.51–0.83). When all 12 measurements were used, the ICC was 0.86 (95% CI 0.78–0.92) (see Table 1), indicating increased reliability with more measurements.

The ICC of the question assessing the ability ‘to perform general computer work’ was found to be 0.71 (95% CI 0.47–0.84) when two repeated measurements were averaged. The ICC showed no increase in reliability for three repeated measurements, but did for twelve repeated measurements [ICC: 0.89 (95% CI 0.84–0.94)]. Test–retest reliability of the question regarding the ability ‘to conduct data analyses’ was 0.76 (95% CI 0.47–0.89) for three repeated measurements and 0.95 (95% CI 0.91–0.98) for twelve repeated measurements (see Table 2).

**Table 2:** Test–retest reliability between two, three or twelve task-specific work ability measurements

Task-specific work ability	ICC (95% CI) 2 measurements	ICC (95% CI) 3 measurements	ICC (95% CI) 12 measurements
To address meetings	0.65 (0.36–081) (n = 44)	0.71 (0.51–0.83) (n = 43)	0.86 (0.78–0.92) (n = 38)
To perform general computer work	0.71 (0.47–0.84) (n = 44)	0.70 (0.50–0.83) (n = 43)	0.89 (0.84–0.94) (n = 38)
To analyse data	0.76 (0.47–0.89) (n = 27)	0.80 (0.62–0.91) (n = 25)	0.95 (0.91–0.98) (n = 20)

### Time-invariant predictors of differences in mean work ability across weeks

The fixed effects (i.e. female, senior researcher, working less than 5 days per week and sufficient leisure time physical activity) improved the model fit compared to the model without these effects (AIC 1391.5 versus 1416.1). Only ‘leisure time physical activity’ showed a statistically significant effect on usual task-specific work ability for ‘addressing meetings’ (*p* = 0.03) and ‘performing general computer work’ (*p* = 0.049). Therefore, we took leisure time physical activity into account when modelling those two aspects of task-specific work ability. Those who were sufficiently physically active in their leisure time reported significantly better mean task-specific work ability, with a difference of about a half unit of the scale of 1–8.

Gender, position and number of working days per week did not have a statistically significant effect on ‘ability to conduct data analyses’ (see Table 3a–c).

**Table 3:** Time-invariant predictors of task-specific work ability to **a** address meetings, **b** perform general computer work, **c** conduct data analyses

Model effects	Estimate	SE	*p*
**a**			
Model for the means*			
Intercept	7.02	0.32	< 0.001
Gender (female)	− 0.05	0.29	0.10
Position (senior researcher)	− 0.30	0.20	0.20
Number of working days (less than 5 days per week)	0.13	0.23	0.60
Leisure time physical activity (sufficient for norm)	0.54	0.25	0.03
**b**			
Model for the means**			
Intercept	6.7	0.38	< 0.001
Gender (female)	− 0.39	0.35	0.28
Position (senior researcher)	− 0.32	0.28	0.26
Number of working days (less than 5 days per week)	0.16	0.28	0.58
Leisure time physical activity (sufficient for norm)	0.60	0.29	< 0.05
**c**			
Model for the means***			
Intercept	6.2	0.57	< 0.001
Gender (female)	− 0.45	0.53	0.40
Position (senior researcher)	− 0.20	0.42	0.63
Number of working days (less than 5 days per week)	0.53	0.43	0.22
Leisure time physical activity (sufficient for norm)	0.61	0.45	0.18

*Model fit: − 2LL = 1377.5; AIC = 1391.5; BIC = 1420.1

**Model fit: − 2LL = 1441.2; AIC = 1455.2; BIC = 1484.0

***Model fit: − 2LL = 979.8; AIC = 993.8; BIC = 1019.9

We calculated that 69% of the variance in task-specific work ability regarding addressing meetings was due to differences in individuals’ usual level of task-specific work ability, and 31% was due to variation in work ability across weeks. Of the 69%, approximately 28% was explained by the effects of leisure time physical activity. The proportional reduction in the random intercept variance relative to the empty means, random intercept model, was 0.28 (pseudo *R* ^2^ = 0.28).

### Time-varying predictors of weekly task-specific work ability

To examine the weekly effects of time-varying predictors on weekly task-specific work ability, we calculated the usual level of work demands and wellbeing of all individuals (usual level as in ‘general’ level or ‘common’ level).

The between-person effect of relational wellbeing on work ability to address meetings was significant (*p* = 0.02) (Table 4a). Participants who reported having 10 scale units lower usual relational wellbeing (on a scale of 0–100) had a 0.6 scale unit lower usual level of work ability for addressing meetings. In addition, we found a statistically significant (*p* = 0.04) effect of personal wellbeing. This indicated that when the participants had 10 scale units lower personal wellbeing than usual, that week’s task-specific work ability to address meetings was lower by 0.2 of a scale unit.

**Table 4:** Time-varying predictors (work demands and wellbeing) of task-specific work ability to **a** address meetings, with leisure time physical activity taken into account, **b** perform general computer work, with leisure time physical activity taken into account, **c** conduct data analyses

Model effects	Estimate	SE	*p*
**a**			
Model for work demands*			
Intercept	6.5	0.15	< 0.001
Leisure time physical activity (sufficient for norm)	0.63	0.25	0.02
Physical demands			
Individual’s usual mean	0.18	0.18	0.32
Demand that week	− 0.03	0.09	0.75
Mental demands			
Individual’s usual mean	− 0.41	0.24	0.09
Demand that week	− 0.09	0.09	0.28
Model for wellbeing**			
Intercept	6.6	0.14	< 0.001
Leisure time physical activity (sufficient for norm)	0.14	0.24	0.56
Personal wellbeing			
Individual’s usual mean	− 0.03	0.04	0.46
Wellbeing that week	0.02	0.007	0.04
Relational wellbeing			
Individual’s usual mean	− 0.06	0.02	0.02
Wellbeing that week	− 0.009	0.008	0.29
Social wellbeing			
Individual’s usual mean	0.04	0.03	0.11
Wellbeing that week	0.003	0.008	0.72
General wellbeing			
Individual’s usual mean	0.07	0.06	0.29
Wellbeing that week	0.04	0.01	< 0.001
**b**			
Model for work demands***			
Intercept	6.3	0.18	< 0.001
Leisure time physical activity (sufficient for norm)	0.66	0.30	0.04
Physical demands			
Individual’s usual mean	0.23	0.22	0.30
Demand that week	− 0.02	0.09	0.83
Mental demands			
Individual’s usual mean	− 0.34	0.28	0.23
Demand that week	− 0.08	0.09	0.36
Model for wellbeing****			
Intercept	6.5	0.17	< 0.001
Leisure time physical activity (sufficient for norm)	0.20	0.29	0.50
Personal wellbeing			
Individual’s usual mean	0.01	0.05	0.79
Wellbeing that week	0.01	0.01	0.15
Relational wellbeing			
Individual’s usual mean	− 0.05	0.03	0.08
Wellbeing that week	0.002	0.01	0.82
Social wellbeing			
Individual’s usual mean	0.07	0.03	0.03
Wellbeing that week	0.01	0.01	0.27
General wellbeing			
Individual’s usual mean	− 0.02	0.08	0.79
Wellbeing that week	0.03	0.01	0.01
**c**			
Model for work demands*****			
Intercept	6.1	0.21	< 0.001
Physical demands			
Individual’s usual mean	0.59	0.33	0.08
Demand that week	− 0.29	0.12	0.01
Mental demands			
Individual’s usual mean	− 0.46	0.39	0.25
Demand that week	− 0.09	0.10	0.38
Model for wellbeing******			
Intercept	6.1	0.21	< 0.001
Personal wellbeing			
Individual’s usual mean	0.01	0.11	0.91
Wellbeing that week	0.01	0.01	0.36
Relational wellbeing			
Individual’s usual mean	− 0.02	0.04	0.64
Wellbeing that week	0.01	0.01	0.31
Social wellbeing			
Individual’s usual mean	0.07	0.05	0.17
Wellbeing that week	0.01	0.01	0.28
General wellbeing			
Individual’s usual mean	− 0.02	0.14	0.89
Wellbeing that week	0.02	0.01	0.08

*Model fit: − 2LL = 1376.9; AIC = 1392.9; BIC = 1425.6

**Model fit: − 2LL = 1292.0; AIC = 1316.0; BIC = 1365.0

***Model fit: − 2LL = 1440.6; AIC = 1456.6; BIC = 1489.5

****Model fit: − 2LL = 1292.0; AIC = 1316.0; BIC = 1365.0

*****Model fit: − 2LL = 971.9; AIC = 985.9; BIC = 1011.9

******Model fit: − 2LL = 952.6; AIC = 974.6; BIC = 1015.6

Concerning task-specific work ability ‘to perform general computer work’, we found that lower social wellbeing was related to lower task-specific work ability (0.07, *p* = 0.03). When the participants reported lower general wellbeing than usual, their task-specific work ability ‘to perform general computer work’ was also statistically significantly lower (0.03, *p* = 0.01).

The effects of both individuals’ usual mental and physical demands and specific weekly mental and physical demands were nonsignificant, with one exception (Table 4c). Work ability to conduct data analyses was 0.30 of a scale unit lower (scale of 1–8) (*p* = 0.01) when that weeks’ physical demands were one scale unit higher (scale of 1–5).

Neither work demands nor wellbeing indicators had statistically significant effects on ‘ability to conduct data analyses’ (Table 4a–c).

## Discussion

Our first conclusion is that there is substantial intra-individual variability in self-reported task-specific work ability as reflected in relatively low ICCs. Secondly, we found that individuals’ leisure time physical activity was related to the average task-specific work ability in relation to two out of three tasks over a 12- week period. Thirdly, we found significant, but rather sparse and inconsistent associations of task-specific work ability with the time-varying predictors. Individual’s average (usual) mean relational and social wellbeing and physical demands and personal and general wellbeing in any specific week were related to a certain task-specific work ability in that week.

Absolute agreement for the questions on task-specific work ability ‘to address meetings’ and ‘to perform general computer work’ was below acceptable for a single measure to use in individual workers, even when the average of twelve measurements was used. Using the average of up to 12 weekly measurements increased the reliability. The high variability in task-specific work ability might not just be a matter of too much random noise. It is plausible that the ability to cope with some types of tasks is naturally highly variable over time. Then, of course, two or three time points are not enough to assess and model change as they do not adequately reflect the true trend. It seems plausible that this phenomenon does not only apply to researchers and is of general importance.

Another useful next step therefore would be to study whether and, if so, how a sufficient level of reliability can be achieved, in such a way that the outcome can be used in individual decision making. When striving for tailored and individualized recommendations and interventions, this is a prerequisite. The need for more sensitive measures is also substantiated by the finding that the smallest detectable change in work ability (on a scale of 1–10) was two points [17]. This is a rather large difference and raises the question whether this measure should be considered too insensitive to use for evaluating interventions, for example [27, 28]. Measuring multiple times might enable a better characterization of work ability and possibly reduce the smallest detectable change. With the present study, we did not expected to measure differences larger than the smallest detectable change from 1 week to another. Using the average of multiple measurements could solve this issue and is likely to become more feasible when modern technological tools, such as mobile applications, are used as research tools.

Furthermore, the participants who were physically active in their leisure time reported significantly better mean task-specific work ability. This finding is in line with previous findings regarding general work ability [19], but was not expected for task-specific workability in researchers.

When physical demands were higher than usual, we found that week’s task-specific work ability to be lower. Previous work found that high physical workload is related to lower work ability [19]. Our results added to this that variation in physical demands within an individual worker also affects task-specific work ability.

Experiences affecting wellbeing also affect task-specific work ability in the short term. Individuals’ with lower mean relational and social wellbeing are expected to have slightly lower task-specific work ability, and lower general or personal wellbeing than usual is expected to affect that specific week’s task-specific work ability. This means that when interpreting work ability values, we should be aware that both fluctuations in work ability and in non-work-related, general factors such as wellbeing co-variate.

Several limitations of our study need to be taken into account. The first is that the study population was limited to only 40 Dutch researchers of an academic medical center. Generalizability to other types of researchers, with different physical demands for example, might therefore not be justified. Secondly, in the present study, we only assessed the ability to address meetings, to perform general computer work and to conduct data analyses. This can be regarded as a too limited view on the specific tasks of researchers. Thirdly, we assumed some predictors to be stable, but for example leisure time physical activity may vary over time. For the purpose of the present study, we did not intend to estimate the amount of physical activity over the weeks. We intended to discriminate those who generally achieve the Dutch standard for healthy physical activity from those who do not generally achieve this standard.

Our findings are a start in understanding the dynamics of assessments of these variables over a short period of time. Similar dynamics, such as momentary feelings and thoughts ‘on the job’ that vary within individuals across time and different job situations at the workplace, have received more attention in recent years [1]. When we acknowledge that outcomes such as work ability are dynamic, the classic approach of characterizing individuals’ work ability as a status is no longer sufficient. We have demonstrated the dynamics of task-specific work ability. Thus, to model aspects of the work ability of individual workers in a better and more sophisticated way, it is crucial to take these dynamics into account. Other aspects of dynamics, such as lag elements, are worth considering as well. New measurement methods, such as using mobile applications, are likely to make it easy to measure multiple times and enable the better characterizing work ability [1].
